# The Effect of Overall Limb Torsion on Functional Femoral Version and Its Functional and Biomechanical Implications on Lower Limb Axial Anatomy: A Study on CT and EOS Imaging

**DOI:** 10.3390/jcm14072448

**Published:** 2025-04-03

**Authors:** Loïc Vercruysse, Michele Palazzuolo, Riza Gultekin, Lachlan Milne

**Affiliations:** 1 Sir Charles Gairdner Hospital, Perth, WA 6009, Australia; michele.palazzuolo@gmail.com (M.P.); lachiemilne@gmail.com (L.M.); 2 Department of Orthopaedic Surgery, KU Leuvern University Hospitals Leuven, 3000 Leuven, Belgium

**Keywords:** functional femoral version, limb torsion, hip arthroplasty, hip preservation

## Abstract

**Background:** Variations in femoral version are increasingly recognized as contributing factors to the development of symptomatic femoroacetabular impingement (FAI) and ischiofemoral impingement (IFI). Despite having implications for both hip arthroplasty and hip preservation surgery, functional femoral version (FFV) and overall limb torsion (OLT) are understudied. This study was conducted with the primary aim of defining and measuring FFV as a function of OLT. **Methods:** A cohort of 106 patients scheduled for primary hip arthroplasty underwent detailed retrospective assessment through CT and EOS imaging. Femoral torsion, transmalleolar axis, tibial torsion, trochanteric station and limb torsion were measured. The trochanteric station distance was also defined on both CT as well as on the lateral standing EOS. Statistical analyses examined the relationships between FFV, OLT, and other measurements. **Results:** Findings indicate a strong correlation between OLT and FFV. Agreement between CT and EOS imaging for trochanteric station was 0.88. **Conclusions:** The study reveals that OLT offers a more comprehensive assessment of impingement risk than anatomical femoral version alone. As OLT correlates with FFV, it highlights the role of axial limb alignment in hip joint biomechanics. Understanding the interplay between FFV and OLT can guide more individualized surgical techniques, potentially improving patient outcomes.

## 1. Introduction

The sagittal plane relationships between the spine and pelvis and its relevance in the field of total hip arthroplasty (THA) is well-documented and understood in recent orthopedic literature [1,2]. The recently emerging literature is thus leading to a paradigm shift in cup positioning, favoring functional positioning over traditional positioning in the Lewinnek safe zone [3]. However, the functional position of the trochanteric station and the functional femoral stem position and its influence on the overall limb torsion (OLT) is understudied, despite its critical role in lower limb function and hip joint biomechanics both in hip arthroplasty and hip preservation setting [4,5,6,7,8,9]. During walking, the foot progression angle (FPA) will influence the position of the trochanter and vice versa, depending on the relationship between the femoral and tibial torsion. In this pilot study, our primary goal was to investigate the relationship between the proximal femur or functional femoral version (FFV) and the distal tibia (TMA), resulting in an angle that we will describe as OLT. This OLT presents a novel area of investigation and the results could suggest a more holistic approach when planning the functional stem position in THA. Retroverting the femoral stem in patients with high anatomical femoral version but with a high tibial torsion could potentially exacerbate the external FPA. Several other studies have demonstrated that native femoral anteversion is patient-specific and should be considered when planning THA. These studies also highlighted the importance of femoral component anteversion as well as anterior femoral offset and showed that increasing femoral anteversion increases both anterior offset and internal rotation of the femur [6,7,8]. Functional femoral anteversion (FFA) was described in a study from Hardwick-Morris et al. and showed a wide range of 80°. It is now understood that the femur, like the pelvis, can rotate substantially between functional positions, and enhancing our understanding of FFA may improve both acetabular and femoral component positioning [10]. The secondary goal is correlating our measurements with both static and functional imaging modalities, such as CT and EOS. This, in turn, may offer significant insights into the management of hip pathology and the optimization of THA outcomes. Preventing common postoperative complications such as early bony or prosthetic impingement and potential secondary instability could lead to improved patient outcomes through more precise and individualized implant positioning.

## 2. Methods

This retrospective cohort study involved 106 patients scheduled for primary hip arthroplasty at our institution. Data collection involved both the CT and EOS ^®^ Imaging techniques to provide comprehensive anatomical and functional assessments. The imaging protocols were consistent across patients. For the CT scan, the acquisition consists of three separate short spiral axial scans with both hips, both knees, and both ankles in a standardized position of the limbs in alignment with their feet positioned anteriorly and without a pillow under the knees. Scans were acquired in slices of minimum 512 × 512 pixels. The thickness of a single slice was no more than 4 mm for the knee and ankle and 1 mm for the pelvis. The spacing between slices was not larger than the slice thickness. The scan ranges for the pelvis start 2 cm above the iliac crest and continue to 15 cm below the lesser trochanter. Both femoral and tibial parts of the knee joint, at least 5 cm on each side, are included in the knee acquisition. The ankle scan includes 5 cm of the tibia and extends past the lateral malleolus. The EOS scan was taken with the feet according to the patient’s comfortable stance. While CT remains the gold standard for static images and axial measurements, EOS imaging provides weight-bearing, full-body images that capture the functional alignment of the lower limbs and pelvis, offering a more dynamic perspective compared to static CT scans. These measurements allowed for detailed analysis of their anatomical relationships within the limb and their contribution to overall alignment and function. CT scans of the pelvis and lower limbs were performed to obtain measurements of femoral version, tibial torsion, and overall limb torsion. These angles were measured by a fellowship-trained arthroplasty surgeon on the transverse plane (original acquisitions). Femoral torsion consists of the addition of the proximal femoral angle (functional femoral version) and the distal femoral angle (posterior condylar axis). Tibial torsion consists of the addition of the proximal tibial angle with the transmalleolar axis (TMA). Femoral torsion was measured according to Murphy, as differences in femoral torsion among various measurement methods increase in hips with excessive femoral torsion as described by Lerch [11]. The trochanteric station distance was also defined on both CT as well as on the lateral standing EOS. On CT it can be defined on the axial slices and on EOS on the sagittal view as the distance between the center of rotation of the femoral head relative to the posterior margin of the greater trochanter measured in mm.

**Figure 1:**
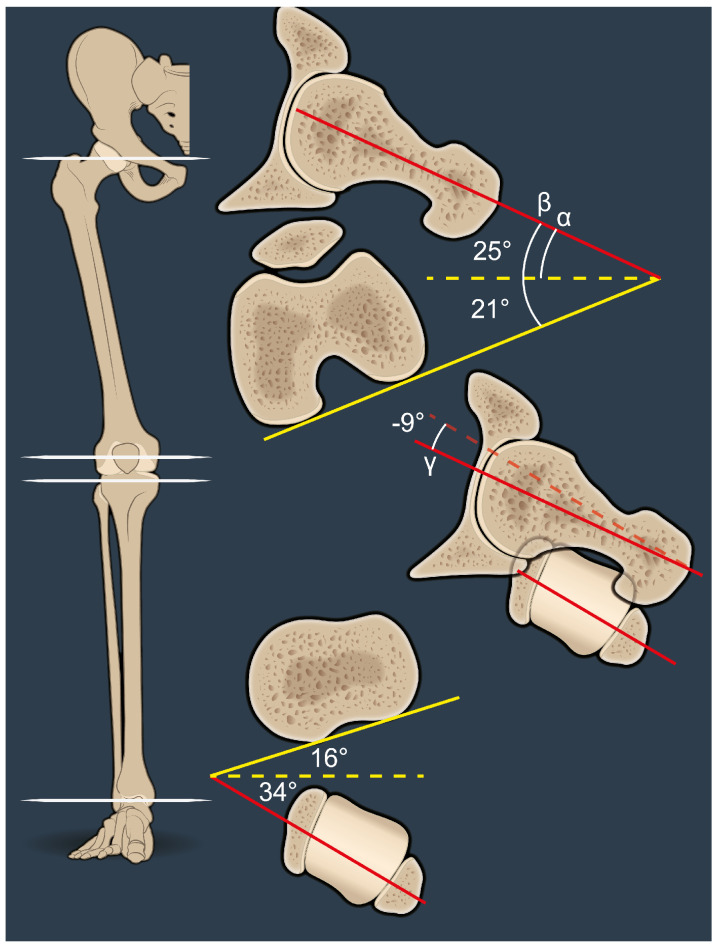
This image shows the anatomical femoral and tibial torsional profiles (red-yellow angles) as well as the relationship between the proximal femur (FFV) and the distal tibia (TMA). This angle describes OLT and is depicted by the Y-angle (red lines). The femoral neck is 9° retroverted relative to the TMA and therefore considered a negative value.

FFV was defined as the angle between the axis of the femoral neck defined according to Murphy (i.e., center of the femoral head and center of the femoral canal at the level of the lesser trochanter) and the coronal plane (alpha angle, Figure 1). This differs from the anatomical femoral version, which is measured relative to the posterior condylar axis (beta angle, Figure 1). OLT was defined as the angle between the axis of the femoral neck—according to Murphy—and the TMA (gamma angle, Figure 1). Positive OLT was defined when the femoral neck is anteverted relative to the TMA, and negative OLT was defined when the femoral neck is retroverted relative to the TMA.

Statistical analyses were performed using the Pearson correlation coefficient to examine the relationships between limb torsion, FFV, and other measured variables. Lin’s Concordance Correlation Coefficient was used to assess the agreement between CT and EOS measurements. A *p*-value of <0.05 was considered statistically significant.

## 3. Results

This retrospective cohort study includes 108 limbs in 106 patients scheduled for primary hip arthroplasty at our institution. There were more females than males (58 females and 48 males) with a mean age of 55.5 years old (range: 26–81; standard deviation: 11.4 years). A significant portion of the females were below the age of 50 years old.

Femoral Parameters: The median proximal femoral angle was 11 degrees (range: −10 to 44 degrees; standard deviation: 9 degrees), and the median distal femoral angle (−ER + IR) was 8 degrees (range: −13 to 32 degrees; standard deviation: 7 degrees). The median femoral version was 20 degrees (range: −4 to 51 degrees; standard deviation: 10 degrees), as measured according to Murphy.

Tibial Parameters: The median proximal tibial angle was 7 degrees (range: −13 to 22 degrees; standard deviation: 6 degrees). The transmalleolar axis had a median value of 24 degrees (range: 2 to 47 degrees; standard deviation: 9 degrees), and the median tibial torsion was 32 degrees (range: 4 to 56 degrees; standard deviation: 9 degrees).

Limb Torsion: The mean overall limb torsion was 12 degrees of retroversion (range: −20 to 45 degrees; standard deviation: 12 degrees), and the median overall limb torsion was 15 degrees of retroversion.

Trochanteric Station: The median trochanteric station was 30 mm as measured by CT (range: 14 to 46 mm; standard deviation: 8 mm) and 30 mm by EOS (range: 12 to 47 mm; standard deviation: 8 mm).

Comprehensive data are presented in Table 1 and Table 2 (Table 1 = CT measurements; Table 2 = EOS measurements).

**Figure 2:**
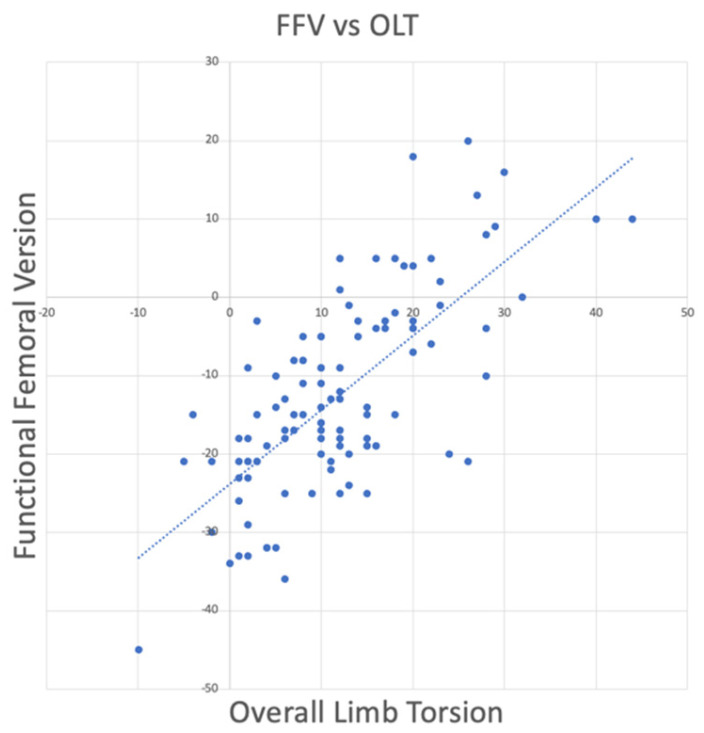
A strong correlation was found between OLT and FFV (Pearson correlation coefficient = −0.65, *p* < 0.001).

**Figure 3:**
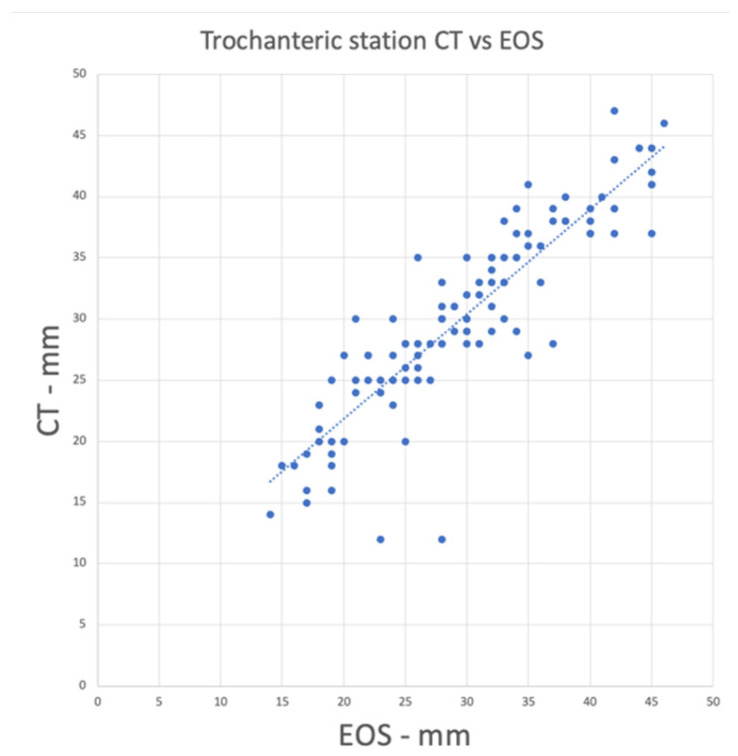
This graph shows a tendency towards agreement between CT and EOS measurements (Lin’s Concordance Correlation Coefficient = 0.88).

A strong correlation was found between OLT and FFV (Pearson correlation coefficient = −0.65, *p* < 0.001, Figure 2). Additionally, the trochanteric station showed a tendency towards agreement between CT and EOS measurements (Lin’s Concordance Correlation Coefficient = 0.88, Figure 3).

## 4. Discussion

The sagittal plane relationship between the spine and pelvis and its relevance in the field of hip replacement is well-documented and understood and has led to the concept of functional cup positioning. Similarly, several studies have highlighted the importance of axial femoral anatomy and femoral torsion both in native hip conditions such as femoroacetabular impingement and developmental dysplasia of the hip, as well as in total hip arthroplasty (THA). These studies, which are mostly done in the field of hip preservation surgery, investigate the importance of femoral and/or tibial derotation osteotomies and its consequences on altered hip biomechanics. However, THA also has the potential to modify both native and functional femoral version, and we therefore aimed to investigate the impact of OLT on FFV (Figure 2) and, consequently, on hip joint biomechanics. The goal of the present study is to depict the relationship between FFV and OLT, which is a novel area of investigation. The relationship between these angles could suggest a more holistic approach when planning the functional stem position in THA. Patients with high anatomical femoral version are at risk for IFI but when FFV is retroverted relative to the TMA, causing negative OLT, retroverting the femoral stem could potentially exacerbate IFI.

The primary finding of this study is that as OLT increases, FFV increases, and vice versa (Figure 2). FFV is represented by the proximal femoral angle, which is different from the anatomical femoral torsion measured according to Murphy. When discussing OLT, it is essential to clearly define the direction considered positive. Specifically, femoral torsion (FT) is typically referenced using the femoral condyles, while tibial torsion (TT) is referenced using the tibia, with the knee as the reference point. If we establish the zero point as the femoral neck relative to the transmalleolar axis, it becomes necessary to explicitly define whether positive values represent anteversion or retroversion of the femoral neck in relation to the TMA. Our proposed approach is to define positive OLT as when the femoral neck is anteverted relative to the TMA and negative OLT as when the femoral neck is retroverted relative to TMA. This convention aligns with the intuitive interpretation that patients with high FT exhibit higher positive limb torsion values, while those with retroversion have more negative values.

An example of a negative OLT of −9° is shown in Figure 1. FFV is retroverted relative to the TMA and it is clear from the angles that OLT is not equivalent to the subtraction of the values between FT and TT. This can only be assumed if the femoral posterior condylar axis and the posterior tibial axis are the same. Similarly, FT only equals the FFV if the distal femoral axis is parallel to the coronal plane (Figure 1).

**Figure 4:**
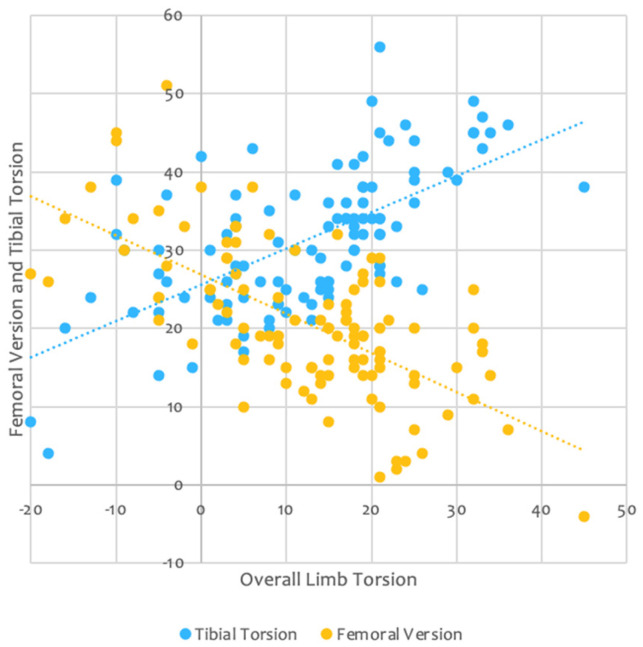
This graph shows that patients with low FT most frequently are accompanied by a high tibial torsion, causing an overall high limb torsion, while those with high FT have a broader distribution of TT values, resulting in low to normal overall limb torsion.

The mean FT in this study was 20° and the mean and median OLT were 12 degrees and 15 degrees of retroversion, respectively. We also found that patients with low FT most frequently have high external TT, while those with high FT have a broad range of external TT, resulting in a spread of OLT, shown in Figure 4. These findings are in concordance with studies by Lerch et al., who have done extensive research on the relationship between FT and TT. Abnormal FT was present in 62% and abnormal TT was present in 42% of their study group. They also reported that the most frequent combination was increased FT combined with normal TT but that the development of FT and TT could also take place independently, as seen in 10% of the patients with dysplasia [12]. We propose that this is due to a disproportionately high number of patients with high FT having high TT, therefore normalizing their FFV and avoiding in-toeing. This was also confirmed in another study of Lerch et al., which found that in-toeing has a low sensitivity to detect high FT [13]. Despite the high prevalence of abnormal FT, clinical diagnosis is challenging.

**Figure 5:**
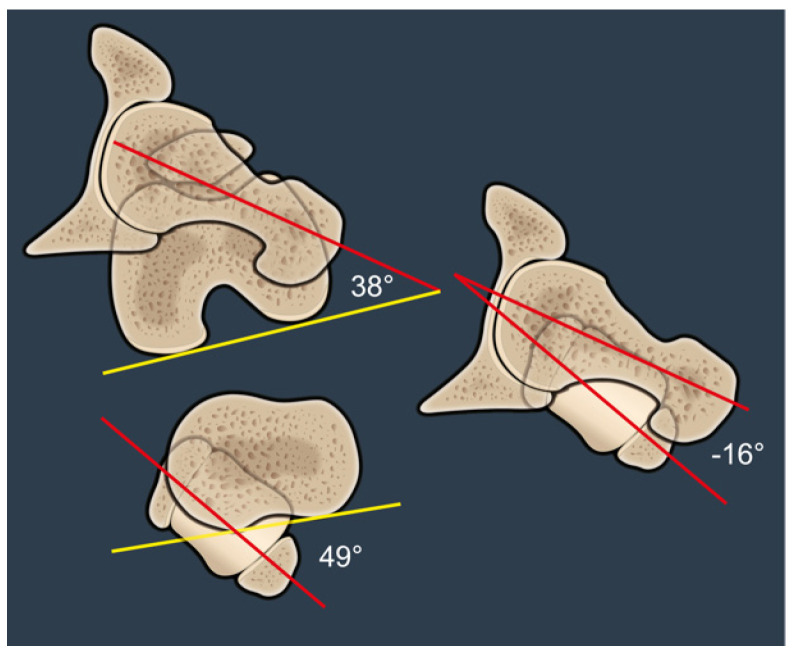
This image shows a negative OLT of 16° (red lines). This patient is therefore at low risk for posterior impingement in hip extension, despite high anatomical anteversion (top red-yellow angle). Correcting this patient’s anteversion in THA will likely result in an externally rotated FPA.

**Figure 6:**
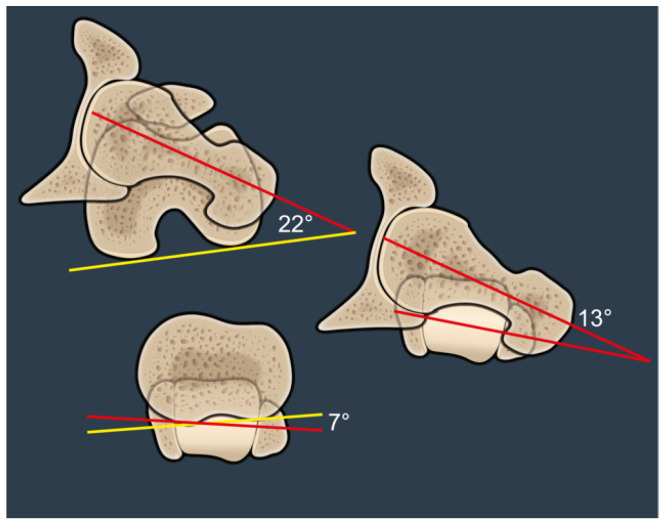
This image shows a positive OLT of 13° (red lines). This patient is therefore at a higher risk for posterior impingement in hip extension and may benefit from a targeted alteration of the femoral stem version (top red-yellow angle) at the time of surgery.

Such an effect on axial limb rotation could play a crucial role in hip mechanics and on the risk of postoperative complications. Moreover, increased OLT may predispose to either intra-articular or extra-articular posterior hip impingement. IFI is a relatively underreported pathomechanical condition characterized by abnormal contact between the ischium and the femur, often due to altered femoral version [4,9,14,15]. Increased femoral version can reduce the ischiofemoral space, leading to impingement and associated symptoms [15]. Our study’s findings on the relationship between OLT and FFV suggest that compensatory mechanisms, such as changes in TT and FPA, may play a role in mitigating the risk of IFI. We will highlight the importance this phenomenon in two cases (Figure 5 and Figure 6) with different OLT and how the TT can dramatically alter OLT and subsequent FFV.

Figure 5 illustrates a patient with a high anatomic FT of 38° and a compensatory high TT of 49°. The FFV is retroverted relative to the TMA and therefore results in a negative OLT of 16°. This patient is therefore at low risk for posterior impingement in hip extension, despite high anatomical anteversion, and correcting this patient’s anteversion in surgery will likely result in an externally rotated FPA.

Figure 6 illustrates a patient with an anatomic FT of 22° and a very low TT of 7°. The FFV is anteverted relative to the TMA and therefore results in a positive OLT of 13°. This patient is therefore at high risk for posterior impingement and may benefit from targeted alteration of femoral version at the time of surgery.

This further highlights the importance of a holistic approach to evaluating limb alignment and particularly functional alignment in patients with hip pathology, especially in patients with excessive femoral version abnormalities who report lower quality of life across all domains measured in the LD-SRS and PROMIS questionnaires [16].

The importance of femoral component anteversion as well as anterior femoral offset was also highlighted in several recent studies that showed that increasing femoral anteversion increases both the anterior offset and internal rotation of the femur, with approximately a 1° increase in internal rotation for every 4° increase in anteversion, on average [6,7,8]. Functional femoral anteversion (FFA) was described in a study from Hardwick-Morris et al. and showed a wide range of 80°: it is thus now understood that the femur, like the pelvis, can rotate substantially between functional positions. Enhancing the understanding of FFA and FFR—based also on limb torsion measurements and axial imaging—may improve both acetabular and femoral component positioning [10,12].

The overall bony alignment described by FFV and OLT, however, does not consider the soft tissue envelope. Pierrepont et al. [7] describe a homeostatic mechanism based on the tension of the anterior and posterior soft tissue structures about the trochanter. They serve to restore the equilibrium to maintain femoral anteversion close to its native value [6]. We believe that the influence of OLT and the FPA will consequently exacerbate or diminish the aforementioned effects of homeostasis, which will help guide surgeons in making decisions about altering femoral version.

The findings from this study have significant implications for the assessment and management of patients undergoing hip arthroplasty and hip preservation surgery. The strong correlation between OLT and FFV suggests that OLT provides a more comprehensive assessment of posterior impingement risk compared to anatomical FT alone, as seen in Figure 4, which illustrates that patients with high FT have a broad range of external TT. This in turn leads to an individualized approach where high FT, which might predispose one to posterior impingement, could be mitigated by a corresponding high TT, thus maintaining overall limb alignment and function. Consequently, there would be no need to change the femoral stem version. Soft tissue compensatory mechanisms, by which the body restores the equilibrium to maintain femoral anteversion close to its native value, can play a role as well [1]. Understanding the complex interplay of axial alignment, biomechanics, and individual anatomical variations can help tailor surgical techniques to individual patient anatomies. Incorporating 3D planning into preoperative assessments could enhance surgical precision and reduce complications such as impingement, edge loading, polyethylene wear, and dislocation, thus optimizing patient outcomes.

A collateral but relevant finding of the actual study is the substantial agreement and consistency between the sagittal functional (EOS) and the axial static (CT) imaging modalities in assessing femoral alignment, as shown in Figure 3. Table 1 (CT) and 2 (EOS) show the distances in mm with a range from 14 to 46 mm and 12 to 47 mm, respectively. On a lateral EOS scan, the distance between the posterior margin of the trochanteric station and the hip center increases when FFV increases. This finding indicates that the trochanteric station is positioned more posteriorly, which may suggest an increased risk of IFI in this patient group. EOS scans depict a high correlation with the CT findings (Figure 3), which suggests it could potentially be utilized in case there is no CT or 3D planning available, but further validation of this correlation is necessary in prospective cohort studies.

This study has several strengths, including its novel exploration of the relationship between FFV and OLT, providing valuable insights into hip biomechanics and its potential to reducing the risk of posterior as well as anterior, bony, and/or prosthetic impingement. It provides a more holistic approach to understanding the axial interplay between the foot and the proximal femur and thus its effect on FFV. Preoperative CT scans with ankle slices and consequently 3D planning are powerful tools in understanding this relationship. The integration of CT and EOS imaging enables a comprehensive analysis of both static and functional alignment. However, this study also has some limitations that should be acknowledged. First, the retrospective design inherently limits the ability to establish causation and may introduce biases related to the collection of historical data. The relatively small sample size, while sufficient for preliminary findings, may not adequately represent broader patient populations or account for anatomical and demographic variations. Additionally, the presence of contralateral hip pathology in some participants could influence the measurements and correlations observed, potentially skewing the results. Although CT and EOS imaging were utilized to capture static and functional alignment, other compensatory mechanisms may impact the assessment of dynamic limb alignment and biomechanics. Furthermore, this study lacks longitudinal follow-up, which limits insights into how functional femoral version and limb torsion evolve over time and how this might impact long-term outcomes. This study does not assess the relationship between OLT values and varying rates of complications, such as impingement and dislocation, as the main goal of this pilot study was to define a relationship between FFV and TMA. Lastly, as this study was conducted in a single institution, the findings may be influenced by local practices and patient demographics, potentially reducing their external validity and generalizability.

These limitations highlight the need for larger, multicenter, and prospective studies to validate the findings and refine clinical applications. Future studies should aim to include larger cohorts, incorporate standing CT scans and dynamic movement assessments to better capture functional limb alignment and its impact on hip biomechanics. Longitudinal studies could also provide valuable insights into how changes in FFV and limb torsion over time influence postoperative outcomes and complication rates. Additionally, further research into the compensatory mechanisms between femoral and tibial torsion could enhance our understanding of both developmental and acquired hip pathologies.

## 5. Conclusions

This study highlights the importance of considering the axial spine–pelvis–lower limb axial anatomy in the assessment and surgical planning for both hip arthroplasty and hip preservation surgery. The interplay between functional femoral version and overall limb torsion underscores the need for a comprehensive approach to evaluating limb alignment. By integrating these measurements into clinical practice, orthopedic surgeons can enhance their understanding of hip pathomechanics, improve surgical outcomes, and reduce the risk of postoperative complications. Further research with larger cohorts and advanced imaging techniques is essential to validate these findings and refine surgical strategies.

## Figures and Tables

**Table 1:** CT measurements.

	Mean	Median	Standard Deviation	Min	Max	Interquartile Range
Proximal femoral angle (degrees)	12	11	9	−10	44	12
Distal femoral angle (−ER + IR) degrees	9	8	7	−13	32	9
Femoral version (degrees)	21	20	10	−4	51	11
Proximal tibial angle (degrees)	7	7	6	−13	22	8
Transmalleolar axis (degrees)	24	24	9	2	47	12
Tibial torsion (degrees)	31	32	9	4	56	12
Overall Limb Torsion (degrees)	12	15	12	−20	45	17
Trochanteric station (mm)	30	30	8	14	46	11
Ischiofemoral distance (mm)	32	32	9	10	54	14
Ischial width (widest point) (mm)	159	159	11	135	185	16
Ischial width relative to hip center (mm)	7	3	9	0	46	10

**Table 2:** EOS measurements.

	Mean	Median	Standard Deviation	Min	Max	Interquartile Range
SVA (Sagittal Vertical Axis) (mm)	13	9	33	−43	129	49
PI (Pelvic Incidence) degrees	55	54	12	32	95	13
PT (Pelvic Tilt) (degrees)	13	13	8	−6	37	11
SS (Sacral Slope) (degrees)	41	42	8	17	68	10
Lordosis (degrees)	61	61	11	28	91	13
Functional Pelvic Plane FPP (degrees)	0	0	6	−14	17	10
Knee Flexion/extension (degrees)	2	2	7	−21	22	7
Pelvic Femoral Angle (degrees)	186	187	11	153	210	12
Functional Femoral Flexion (degrees)	7	7	5	0	21	9
Trochanteric Station (mm)	30	30	8	12	47	11

## Data Availability

No new data were created or analyzed in this study. Data sharing is not applicable to this article.
